# Modulating Pharmacokinetics, Tumor Uptake and Biodistribution by Engineered Nanoparticles

**DOI:** 10.1371/journal.pone.0024374

**Published:** 2011-09-13

**Authors:** Rochelle R. Arvizo, Oscar R. Miranda, Daniel F. Moyano, Chad A. Walden, Karuna Giri, Resham Bhattacharya, J. David Robertson, Vincent M. Rotello, Joel M. Reid, Priyabrata Mukherjee

**Affiliations:** 1 Department of Biochemistry and Molecular Biology, Mayo Clinic Rochester, Rochester, Minnesota, United States of America; 2 Department of Chemistry, University of Massachusetts, Amherst, Massachusetts, United States of America; 3 Department of Oncology, Mayo Clinic Rochester, Rochester, Minnesota, United States of America; 4 Department of Physiology and Biomedical Engineering, Mayo Clinic Rochester, Rochester, Minnesota, United States of America; 5 Department of Chemistry and University of Missouri Research Reactor, University of Missouri, Columbia, Missouri, United States of America; 6 Mayo Clinic Cancer Center, Mayo Clinic College of Medicine, Rochester, Minnesota, United States of America; Ohio State University, United States of America

## Abstract

**Background:**

Inorganic nanoparticles provide promising tools for biomedical applications including detection, diagnosis and therapy. While surface properties such as charge are expected to play an important role in their *in vivo* behavior, very little is known how the surface chemistry of nanoparticles influences their pharmacokinetics, tumor uptake, and biodistribution.

**Method/Principal Findings:**

Using a family of structurally homologous nanoparticles we have investigated how pharmacological properties including tumor uptake and biodistribution are influenced by surface charge using neutral (**TEGOH**), zwitterionic (**Tzwit**), negative (**TCOOH**) and positive (**TTMA**) nanoparticles. Nanoparticles were injected into mice (normal and athymic) either in the tail vein or into the peritoneum.

**Conclusion:**

Neutral and zwitterionic nanoparticles demonstrated longer circulation time via both IP and IV administration, whereas negatively and positively charged nanoparticles possessed relatively short half-lives. These pharmacological characteristics were reflected on the tumor uptake and biodistribution of the respective nanoparticles, with enhanced tumor uptake by neutral and zwitterionic nanoparticles via passive targeting.

## Introduction

The limited solubility, stability, and short circulation time of traditional therapeutic agents can introduce side effects and unwanted accumulation of therapeutics into non-diseased tissue [1]. Multimodal nanoparticles, polymer-drug conjugates, monoclonal antibodies and immunoconjugates provide a means of increasing therapeutic efficacy [2], while also providing new therapeutic and imaging modalities [3]. The diversity of available structures and core materials [4], [5] coupled with sub-cellular size and biocompatibility have generated numerous applications of these materials in fields ranging from delivery [6]–[8] and imaging [9], to photothermal ablation of tumors [10], [11].

Key issues for the creation of effective nanotherapeutics include (i) overcoming biological barriers, (ii) specific accumulation of the therapeutic at the target site (i.e, targeting), and (iii) preventing rapid clearance [12]. While there have been a number of studies on the effects of particle size on biodistribution [13], little is known about the effect of surface charge on biodistribution and pharmacokinetics of nanoparticles, as these studies have generally used neutral poly(ethylene glycol) (PEG) functionalization [14]. This “stealth” coverage decreases the rate of opsonization, providing more efficient transport to the target tissue, e.g. tumor. The neutral coating provided by PEG coverage provides one route to enhanced bioavailability. Charged systems including zwitterionic surfaces [15], however, provide an alternative means of dictating the bioavailability of nanomaterials. To date, there has not been a detailed statistically robust investigation on how the surface charge of nanosystems impact their biodistribution, pharmacokinetics and tumor uptake.

In the current study, we systematically investigate the role of the surface charge of engineered gold nanoparticles in dictating the pharmacokinetics, and hence the biodistribution and tumor uptake of these nanomaterials. As the biodistribution of drug carriers is dependent upon the route of administration [16], we administered these particles using both intravenous (IV) and intraperitoneal (IP) routes [17]. We first assessed how surface charge impacts pharmacokinetics (PK) and biodistribution of the particles in a mouse model, demonstrating that surface charge determines circulation time of gold nanoparticles in the blood and tumor uptake through passive targeting. Through these studies, we reveal that surface charge and mode of administration has tremendous consequences on nanoparticle behavior *in vivo*, thus providing insight for the improved design in nanoparticle therapeutics.

## Results

### Nanoparticle Fabrication

**Figure 1:**
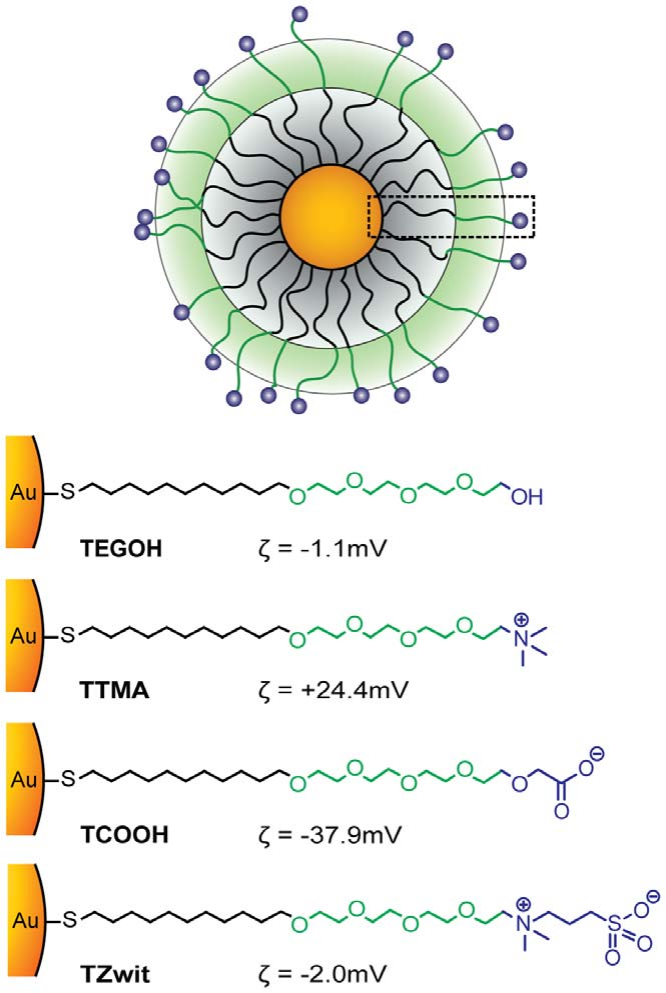
Structural representation of gold nanoparticles (2 nm core diameter) used. Gold nanoparticles (AuNPs) of different surface charges were generated by chemical modification of the terminal portion of the ligand bonded to the nanoparticle core. Four types of AuNPs were used neutral (TEGOH), positive (TTMA), negative (TCOOH) and zwitterionic (TZwit). The surface charge was measured by zeta potential.

Our studies used gold nanoparticles (AuNP) featuring 2 nm diameter cores, with overall hydrodynamic diameters of ∼9–10 nm. As shown in Figure 1, all nanoparticles contain a hydrophobic interior that confers stability [18] and a tetra(ethylene glycol) functionality to provide compatibility and solubility in cell culture media and other biological fluids. The interactions of these particles are dictated by the choice of headgroups, facilitating the determination of structure-activity correlations [19]. These particles are also exceptionally stable in biofluids [20], rendering them useful as drug delivery vehicles [21] and in sensing [22]. To provide a concise study of the role of charge in bioavailability, AuNPs featuring anionic, cationic and zwitterionic particle surfaces were fabricated using ligands having the appropriate headgroups [23]. These particles have been shown to be stable against aggregation in serum and have relatively low toxicities in both *in vitro* cell assays [24] and in fish [25]. Central to our studies, the common structure shared by these particles allows direct assessment of the role of charge on *in vivo* behavior (see Figure S1).

### Plasma Pharmacokinetics is affected by nanoparticle surface charge

**Figure 2:**
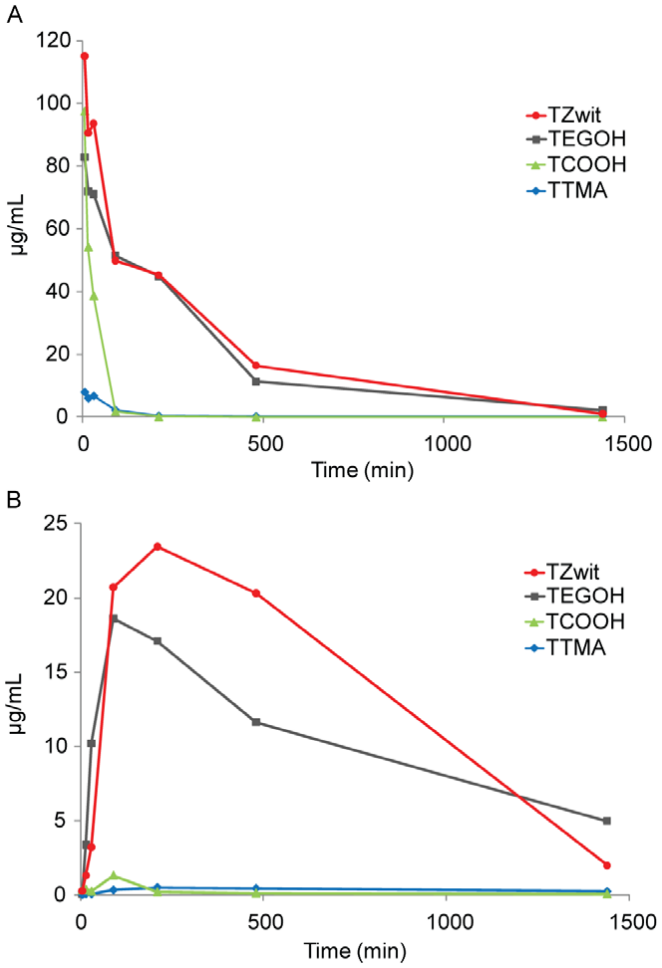
Plasma profiles for gold nanoparticles. Normal mice were injected either (A) intravenously or (B) intraperitoneally. Data points are the mean +/− SEM from n = 3 animals.

The plasma dispositions of the AuNPs were characterized in male CD1 mice after IV and IP administration. Plasma concentration-time profiles for each AuNP are illustrated in Figure 2, with the pharmacokinetic parameters are summarized in Table 1. High peak plasma concentrations (Cmax) >80 mg/ml were achieved with IV injection of the negative (**TCOOH**), neutral (**TEGOH**) and zwitterionic (**TZwit**) particles, while a 10-fold lower peak plasma concentration was achieved with the positive (**TTMA**) particles (Table 1). Following IV injection, high plasma clearance was observed for the negative (**TCOOH**) and positive (**TTMA**) charged AuNPs (0.0739 µg/ml/min and 0.170 µg/ml/min, respectively) such that the plasma concentrations fell below 1 mg/ml within 15 minutes after injection (Figure 1A). In contrast, plasma clearance for the neutral (**TEGOH**) and zwitterionic (**TZwit**) particles was substantially lower (0.00605 µg/ml/min and 0.00561 µg/ml/min, respectively) such that plasma concentrations remained above 1 µg/ml 24 hours after injection (Figure 1A). After IP injection, low concentrations (<1 µg/ml) of the negative (**TCOOH**) and positive (**TTMA**) AuNPs were detected in the circulation (Figure 2B). In marked contrast, the neutral (**TEGOH**) and zwitterionic (**TZwit**) AuNPs rapidly entered into circulation, with peak concentrations above 10 µg/ml achieved 1.5 and 3 hours, respectively. As with the IV injection, plasma concentrations of the neutral (**TEGOH**) and zwitterionic (**TZwit**) AuNPs remained above 1 µg/ml 24 hours after injection (Figure 2B) and bioavailability values for these AuNP were high (77% and 70%, respectively). From these results, it is apparent that nanoconjugates with neutral and zwitterionic properties maximize the circulation time.

**Table 1:** AuNP Pharmacokinetics Summary.

	IV				IP			
	TEGOH	TTMA	TCOOH	TZwit	TEGOH	TTMA	TCOOH	TZwit
C_max_ (µg/ml)	82.93	7.88	97.48	115.13	18.61	0.50	1.32	23.45
T_max_ (min)	5	5	5	5	90	210	90	210
t_1/2_ (min)	304	1428	18	229	784	1079	1178	287
AUC_0-∞_ (mg/ml*min)	2.65E04	1.02E03	3.03E03	2.98E04	2.65E04	1.02E03	3.03E03	2.98E04
CL_p_ (ml/min)	6.05E-03	1.70E-01	7.38E-02	5.60E-03				
F (%)†					78	86	14	70

Dose (mg): TEGOH 160, TTMA 174, TCOOH 224, TZwit 167.

† F = AUC_i.p._/AUC_i.v._×100; C_max_ = Peak plasma concentration; CL_p_ (ml/min) = Plasma clearance; T_max_ = Time.

### Tumor Uptake of AuNPs is related to circulation resident time

**Figure 3:**
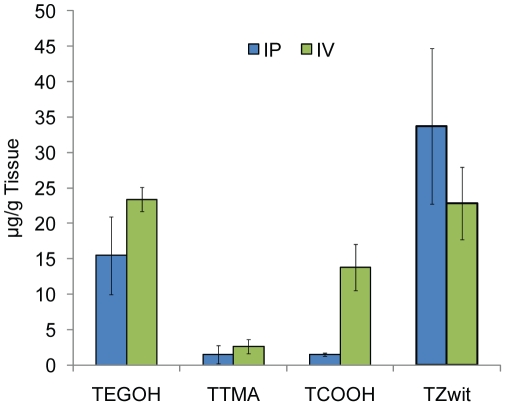
Quantification of *in vivo* accumulation of gold nanoparticles into tumors. Coinciding with the blood concentration, nanoparticles that showed a long retention time in circulation were able to extravasate and accumulate into the tumor. Data points are the mean +/− SEM from n = 5 animals.

Passive targeting of nanomaterials via the enhanced permeability and retention (EPR) effect is dependent on their blood resident time [26]. Our hypothesis was that the neutral and zwitterionic nanoconjugates would exhibit increased tumor uptake relative to negative and positively charged analogs. As expected, the nanoparticles with a long retention time in circulation (**TEGOH** and **TZwit**) accumulated more efficiently into the tumor (Figure 3). After 24 hours post-injection (200 µg per mouse via tail vein or intraperitoneal), subcutaneously implanted ovarian tumor-bearing athymic nude mice were euthanized and the tumors were analyzed for gold content. The **TZwit** and **TEGOH** nanoparticles had substantially higher tumor uptake irrespective of the mode of administration. When injected into the peritoneum, a minute amount of the charged particles accumulated in the tumor, as expected from our pharmacokinetic studies. However, the negative nanoparticle **TCOOH** accumulated in the tumor after IV injection whereas the positive particle **TTMA** did not. These results are consistent with the pharmacokinetics of the IV administered nanoparticles. Thus, as a result of improved pharmacokinetics, neutral and zwitterionic particles demonstrated increased tumor accumulation with both routes of administration as compared to the other nanoparticles through the EPR (Enhanced Permeability and Retention) effect [27]. Our studies are in agreement with previous reports indicating that long plasma half-life can lead to increased tumor uptake [28], an important goal in imaging, diagnostics and therapeutics [29].

### Ligand end group influences biodistribution of AuNPs

**Figure 4:**
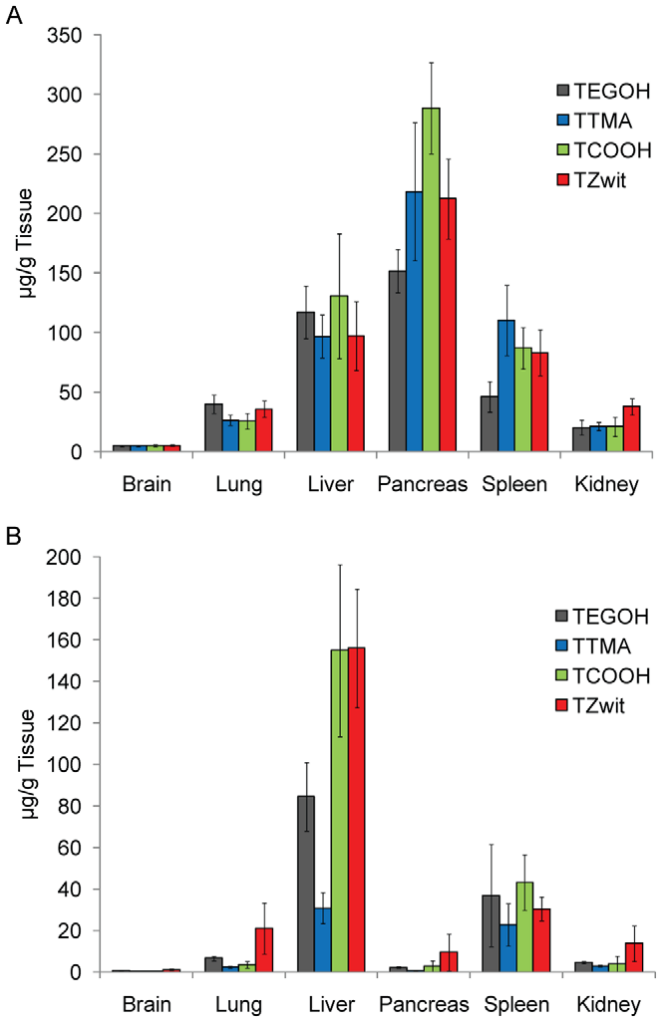
Tissue distribution of gold nanoparticles in mice. *In vivo* mean gold concentration (µg) per gram of organ 24 hours post (A) IP injection and (B) IV injection. The mode of administration and the ligand end group of AuNPs affects the level of gold uptake in different tissues with the RES being the dominant mode of clearance. Data points are the mean +/− SEM from n = 5 animals. Results are reported as gold concentration (µg) per gram of organ.

To determine the role of particle charge in regulating the biodistribution of these engineered nanomaterials, we compared the organ distribution of the AuNPs in immunocompetent mice (IV vs. IP, Table 2). After IV injection the nanoparticles were predominantly localized in the liver and spleen (Figure 4B), with little particle found in the brain, kidneys, or lungs. In contrast, 24 hours after IP injection the concentration of gold was the highest in the pancreas (Figure 4A) for all four particles. This localization may arise from intraperitoneal circulation and altered lymphatic clearance [30]; nanoparticles are removed from systemic circulation through the permeable vascular endothelium in lymph nodes [27], [31]. The difference in localization demonstrates that the mode of administration of AuNPs affects the level of particle uptake in different tissues. Moreover, the levels of AuNPs found in the liver and spleen indicate that RES is the dominant mode of clearance for these particles.

**Table 2:** Biodistribution Study.

	Brain (µg/g)		Lung (µg/g)		Liver (µg/g)		Pancreas (µg/g)		Spleen (µg/g)		Kidney (µg/g)	
	IP	IV	IP	IV	IP	IV	IP	IV	IP	IV	IP	IV
TEGOH	4.8	0.441	39.9	6.471	117.2	84.5	151.6	2.063	46.1	36.8	20.16	4.5
TTMA	4.7	0.268	26.4	2.339	96.6	30.8	218.2	0.660	110.2	22.7	21.04	2.8
TCOOH	4.8	0.174	25.5	32.737	130.7	154.8	288.4	2.632	86.9	43.1	21.08	3.6
TZwit	5.2	1.070	35.6	20.926	97.3	156.0	212.2	9.334	82.9	30.4	37.88	13.7

Dose (mg): TEGOH 160, TTMA 174, TCOOH 224, TZwit 167.

## Discussion

In recent years, inorganic nanoparticles have emerged as drug delivery systems [32], imaging agents [29] and diagnostic biosensors [33]. However, the biological fate and effect of nanoparticles in living systems needs to be defined in order to improve therapeutic engineering for *in vivo* applications, including passively [34] and actively targeted vectors [35]. Previous work in this field has primarily focused on the biodistribution of inorganic nanoparticles based on their size. In our studies we maintained a constant particle size while varying the surface properties of the nanoparticle (see Figure S1, S2, and S3). Using pegylated gold nanoparticles, we investigated the pharmacokinetics, tumor uptake, and biodistribution of 2 nm diameter core/10 nm overall (Figure 1) particles, focusing on the effect of surface charge on *in vivo* behavior. Blood sampling revealed that the neutral and zwitterionic particles **TEGOH** and **TZwit** AuNPs provided the highest concentrations and IP bioavailability. These concentrations were sustained over the 24-hour study period. The particle pharmacokinetics after IP injection was likewise strongly dependent upon particle surface charge. Plasma clearance was very low in comparison to the charged AuNPs (Figure 2). This marked difference in bioavailability could be primarily due to opsonization of the nanoparticles with antibodies for recognition by resident macrophages. With the high concentration of phagocytes in the RES, opsonized particles are cleared out within minutes. Previous reports have also indicated that positively charged polymeric and hybrid nanoparticles accumulate in the liver and spleen in spite of PEG functionalization [36], [37]. However, we show by modifying the nanoparticle surface charge, the pharmacokinetics of the engineered nanoparticles can be tuned.

One of the major issues facing pharmacological agents is tissue selectivity [38]. Since the window of delivery of therapeutic agents is often limited it is imperative that penetration into highly vascular tumors is efficient. Accumulation in the tumor environment determines the therapeutic efficacy of antitumor therapeutics. However, this accumulation is diminished by non-specific tissue uptake and clearance by the RES. By utilizing the EPR effect, it is possible for long-circulating nanoparticles to reach and accumulate in the tumor [27]. In our studies, the more bioavailable **TEGOH** and **TZwit** nanoparticles exhibited a significantly tumor uptake over the **TTMA** particles after IV injection. Futhermore, with IV injections, **TCOOH** along with the **TTMA** and **TEGOH** nanoparticles had increased tumor uptake compared to the **TTMA** nanoparticle. Previous reports have also observed that a long plasma half-life can lead to increased tumor uptake [14], enabling biomedical applications such as imaging, diagnostic and therapeutics.

While all of the nanoparticles used in this study had a pronounced uptake in the liver following IP injection, it is interesting to note that when injected through the tail vein, **TCOOH** and **TZwit** uptake in the liver was considerable compared to the other nanoparticles. Previous reports have shown that β-2 glycoprotein and apolipoprotein B are known to bind to negatively-charged surfaces [39]. These proteins attach to foreign substances forming lipoproteins and transport them though the body via the lymphatic system or blood. Since both proteins are hepatic, it is possible that the **TCOOH** and **TZwit** nanoparticles are being sequestered to the liver due to the negative entity of their headgroup. Another interesting point is that IP injection resulted in high levels of particles in the pancreas. This localization may arise from intraperitoneal circulation and altered lymphatic clearance [30]; nanoparticles are removed from systemic circulation through the permeable vascular endothelium in lymph nodes [27], [31].

In conclusion, charge is a key determinant of the interactions of biological and synthetic materials with biosystems. We have quantified the role of surface charge on pharmacokinetics, tumor uptake and biodistribution using a structurally consistent family of neutral, zwitterionic, negative and positive gold nanoparticles (2 nm core, 10 nm overall diameter, see Figures S1 and S2). Neutral and zwitterionic particles provide high systemic exposure and low clearance when administered through intravenous administration and are rapidly absorbed in the circulation after intraperitoneal administration. Negative particles provide moderate systemic exposure while positive particles are rapidly cleared. Both positive and negative particles are poorly absorbed in the circulation after intraperitoneal administration, indicating the inability of these particles to cross the peritoneal barrier. Low plasma clearance for both administration routes is reflected in the increased tumor uptake of the neutral and zwitterionic nanoparticles in a subcutaneously implanted xenograft model of ovarian cancer. Biodistribution studies in different mouse strains (immunodeficient vs. immunocompetent) demonstrate that surface charge of the nanoparticles and their modes of systemic administration uniquely alter their pharmacokinetics, organ distribution and tumor uptake. The ability of surface charge to dictate bioavailability provides critical information to improve the design of nanotherapeutics for enhanced tumor uptake by both passive and active targeting strategies.

## Materials and Methods

### Gold nanoparticle synthesis and characterization

The Brust-Schiffrin two-phase synthesis method [40], [41] was used for synthesis of AuNPs with core diameters around 2 nm (Figure S1). After that, Murray place-exchange method was used to obtain functionalized AuNPs (Figure S3) [22], [42].

### Animals

All experiments were done under protocols approved by the Mayo Clinic Institutional Animal Care and Use Committee (Protocol No A14108). Male HEJ/C3H mice, 4–6 weeks old and athymic mice (4–6 week old) were obtained from NCI Repository (Fredrick, MD). Male CD1 mice (∼20 g) were received from Charles River.

### Pharmacokinetics

AuNPs (100 µL of 40 µM) were administered to male CD1 mice (20 g) intravenously (IV) via the lateral tail vein and intraperitoneally (IP) in the right side of the stomach using a tuberculin syringe fitted with a 27-gauge needle. Blood samples were collected 5, 15, 30, 60, 90, 210, 480 and 1440 min post-injection. Mice were anesthetized under isoflurane vapors and blood samples collected by cardiac puncture using a 10% heparin in citrate phosphate dextrose solution anticoagulant (150 µL anticoagulant/ml whole blood), transferred to silanized amber microcentrifuge tubes and immediately chilled on ice. After separation by centrifugation (10,000 rpm ×3 min at 4°C) plasma was transferred to silanized amber microcentrifuge tubes and immediately frozen. Samples were stored at −70°C until analysis. AuNP plasma concentration–time data were analyzed by standard noncompartmental methods using the program WinNonlin Pro (Pharsight Corp, Mountain View, CA).

### Cell Culture

CP-70 cells were grown and maintained in RPMI medium supplemented with 10% fetal bovine serum and 1% antibiotic-antimycotic. Cultures were maintained at 37°C and 5% CO_2_ atmosphere.

### Tumor Xenograft

The animal use protocol was approved by the Mayo Clinic Animal Care and Use Committee (IACUC). CP-70 cells were lifted from tissue culture dishes using trypsin (1×) and pelleted at 1500 rpm. Cells were resuspended in HBSS and counted using a hemocytometer. A 100 µL volume containing 2×10^6^ cells was injected subcutaneously on the right flank of 4–6 week old male athymic mice. Tumor growth was monitored until the mass reached 0.5 cm in length in any direction.

### Biodistribution Study

The mice were randomized prior to nanoparticle injection. Mice were injected with 200 µg of AuNPs via IP or IV injections. After 24 hours, the mice were euthanized by CO_2_ asphyxiation and tissue was collected for analysis. Total uptake of gold was analyzed using Instrumental Neutron Activation Analysis (INAA) [43].

## Supporting Information

Figure S1 **TEM images of the gold nanoparticles used in the study.**
(TIF)

Figure S2 **DLS measurements of nanoparticle size (1 uM of AuNPs in PB 5 mM, pH 7.4).**
(TIF)

Figure S3 **Charge distribution of the functionalized gold nanoparticles used in the study (1 uM of AuNPs in 5 mM PBS, pH 7.4).**
(TIF)
