# Supplementary figures and images for: Modulating Pharmacokinetics, Tumor Uptake and Biodistribution by Engineered Nanoparticles
Source: PLoS One. 2011 Sep 13;6(9):e24374. doi: 10.1371/journal.pone.0024374 (PMC3172229; doi:10.1371/journal.pone.0024374)

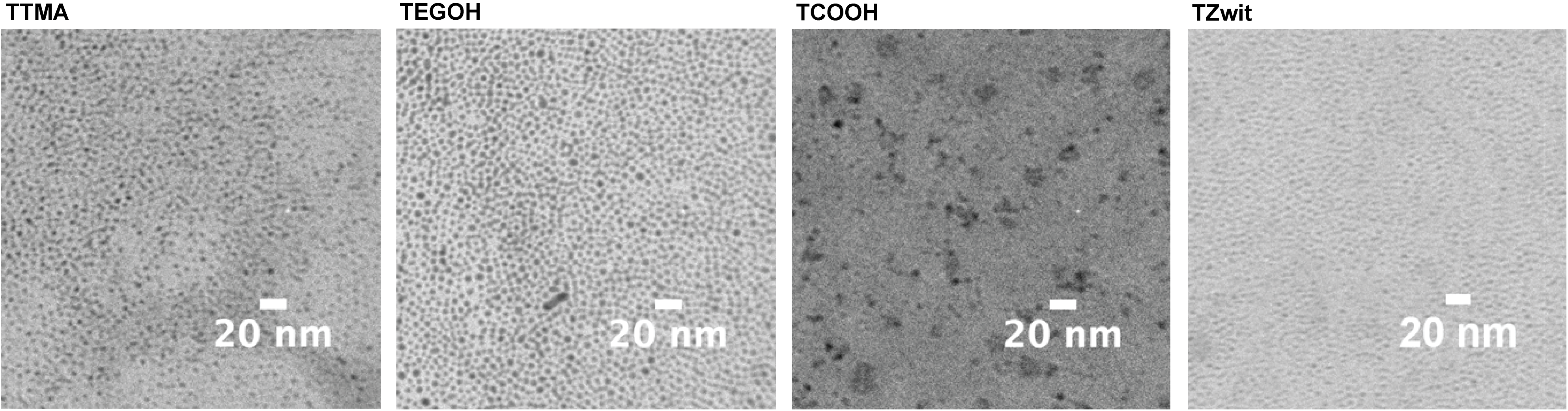

Supplement: Figure S1 — TEM images of the gold nanoparticles used in the study. (TIF) [file pone.0024374.s001.tif]

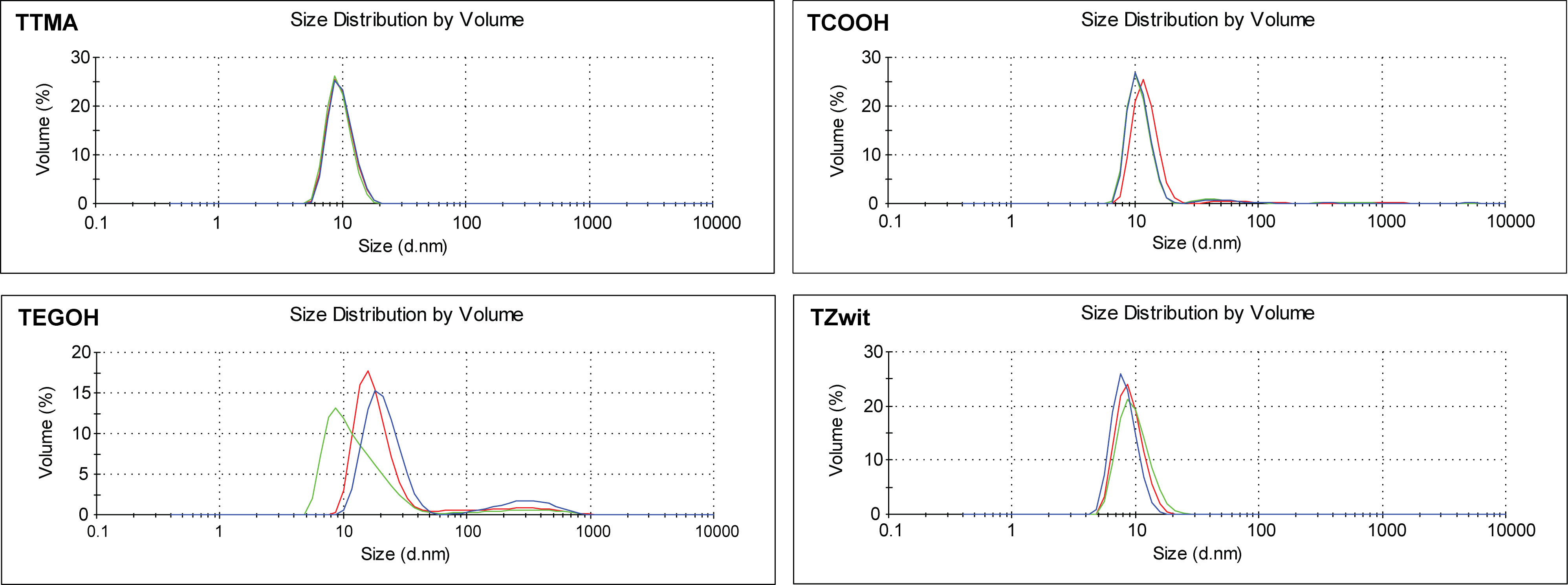

Supplement: Figure S2 — DLS measurements of nanoparticle size (1 uM of AuNPs in PB 5 mM, pH 7.4). (TIF) [file pone.0024374.s002.tif]

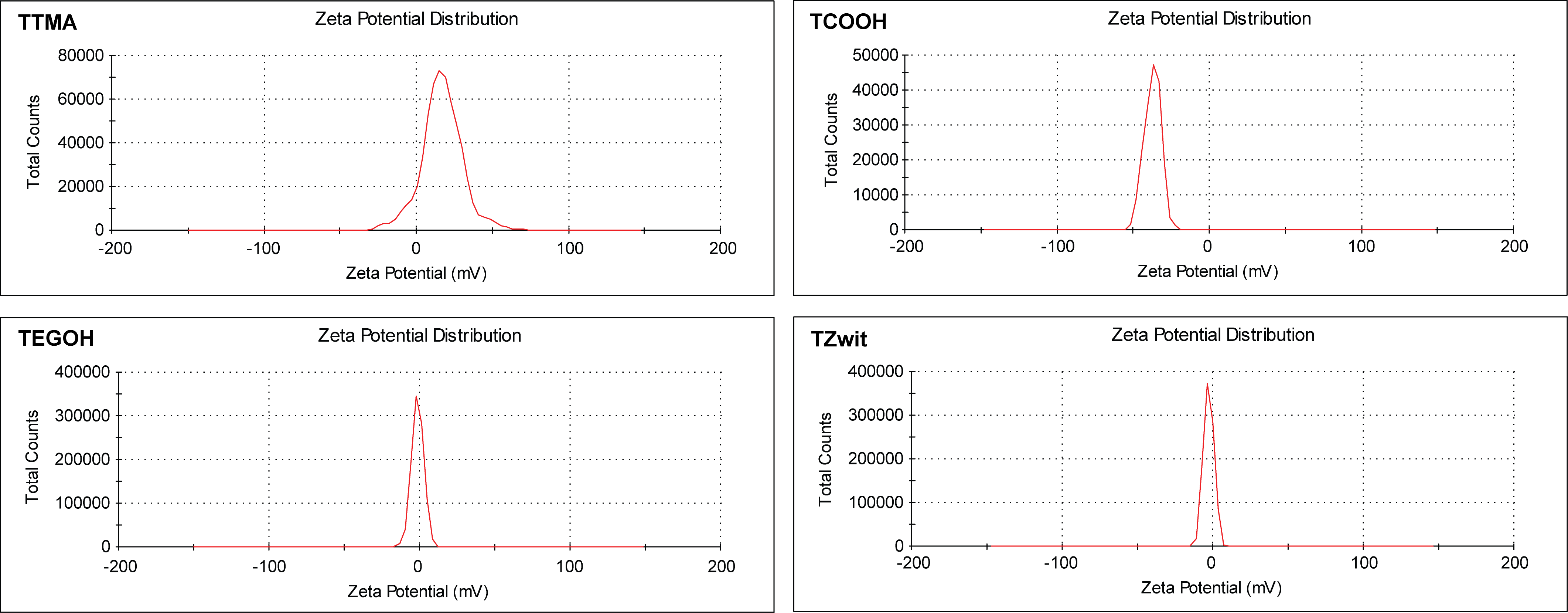

Supplement: Figure S3 — Charge distribution of the functionalized gold nanoparticles used in the study (1 uM of AuNPs in 5 mM PBS, pH 7.4). (TIF) [file pone.0024374.s003.tif]
